# Can clade age alone explain the relationship between body size and diversity?

**DOI:** 10.1098/rsfs.2011.0075

**Published:** 2012-02-01

**Authors:** Rampal S. Etienne, Sara N. de Visser, Thijs Janzen, Jeanine L. Olsen, Han Olff, James Rosindell

**Affiliations:** 1 Community and Conservation Ecology, Centre for Ecological and Evolutionary Studies, University of Groningen, PO Box 11103, 9700 Groningen, The Netherlands; 2 Marine Benthic Ecology and Evolution, Centre for Ecological and Evolutionary Studies, University of Groningen, PO Box 11103, 9700 Groningen, The Netherlands; 3 Faculty of Biological Sciences, Institute of Integrative and Comparative Biology, University of Leeds, Leeds LS2 9JT, UK

**Keywords:** birth–death model, diversification, stochastic model, cladogenesis, maximum likelihood

## Abstract

One of the most striking patterns observed among animals is that smaller-bodied taxa are generally much more diverse than larger-bodied taxa. This observation seems to be explained by the mere fact that smaller-bodied taxa tend to have an older evolutionary origin and have therefore had more time to diversify. A few studies, based on the prevailing null model of diversification (i.e. the stochastic constant-rate birth–death model), have suggested that this is indeed the correct explanation, and body-size dependence of speciation and extinction rates does not play a role. However, there are several potential shortcomings to these studies: a suboptimal statistical procedure and a relatively narrow range of body sizes in the analysed data. Here, we present a more coherent statistical approach, maximizing the likelihood of the constant-rate birth–death model with allometric scaling of speciation and extinction rates, given data on extant diversity, clade age and average body size in each clade. We applied our method to a dataset compiled from the literature that includes a wide range of Metazoan taxa (range from midges to elephants). We find that the higher diversity among small animals is indeed, partly, caused by higher clade age. However, it is also partly caused by the body-size dependence of speciation and extinction rates. We find that both the speciation rate and extinction rate decrease with body size such that the net diversification rate is close to 0. Even more interestingly, the allometric scaling exponent of speciation and extinction rates is approximately −0.25, which implies that the *per generation* speciation and extinction rates are *independent* of body size. This suggests that the observed relationship between diversity and body size pattern can be explained by clade age alone, but only if clade age is measured in generations rather than years. Thus, we argue that the most parsimonious explanation for the observation that smaller-bodied taxa are more diverse is that their evolutionary clock ticks faster.

## 1. Introduction

The predominance of small animals across the tree of life is among the earliest macro-ecological observations [1–3] and continues to intrigue biologists (reviews in [4–6]). The classic macro-evolutionary explanation for this phenomenon is that small animals show higher diversification rates, either due to higher speciation/origination [7–9]—or due to lower extinction rates [7,10] or both. Body size is associated with several evolutionarily important life-history traits that make such an explanation plausible. Large-bodied animals are thought to have lower speciation rates because they generally have longer generation times [11], lower reproductive rates (owing to smaller litter size [12] and longer gestation times [13]), lower mutation rates (because of a lower per mass metabolic rate; [14]) and smaller chance for an adaptive mutation to occur (owing to lower density/smaller population size [2,3]). However, it has also been argued that small population size can actually increase speciation rate through drift and selectively advantageous founder effects [15–17]. Larger animals are more prone to extinction [7,10,18–20], because of their lower birth rates, higher requirement of resources and energy [8,21], larger home ranges [18] and smaller geographical range size [22], making them more vulnerable to environmental disturbances [23]. Lower speciation rates and higher extinction rates in larger animals imply lower net diversification rates, i.e. the net difference between speciation and extinction rates [24]. Thus, there is sufficient reason to quantitatively study the effects of body size on speciation and extinction rates.

However, studies that directly address the relationship between net diversification rate and body size suggest that there is no significant relationship [12,25–27]. The observed larger diversity in smaller-bodied taxa is rather attributed to their older evolutionary age [20,26–28]: smaller-bodied taxa are for some reason (perhaps a taxonomic artefact) evolutionary older, which gave them more time to diversify. Nee *et al.* [25] did find some remaining body-size dependence, but attributed this to phylogenetic non-independence.

Yet, there are several reasons to revisit the question whether clade age alone can explain the relationship between diversity and body size. First, studies exploring a macro-evolutionary explanation for this relationship are scarce. Second, they are based on a limited selection of taxa (e.g. only mammals or birds, or animals on a single continent), namely those for which phylogenetic or fossil data exist. Third, inferences based solely on phylogenies may be flawed [29,30], and the fossil record has limitations as well, e.g. incompleteness and size bias [31–33] (but see [34,35]). Using only the extant number of species in a clade seems more reliable. Last, but not least, the statistical approach to analyse these data is not optimal. The general procedure is as usually as follows: speciation and extinction rates are estimated separately for each clade, log-transformed, and then regressed against the logarithm of body size (because biological rates are generally power-laws of body size, [36]). This procedure has three drawbacks: first, it involves two separate statistical methods (estimation of the rates on the one hand and regression on the other). Second, it cannot deal with clades having only one species (when phylogenies are used, the number of species is typically larger than two), and the estimate of the net diversification rate becomes 0 preventing logarithmic transformation. Third, the approach does not correct for the bias arising from the fact that only clades of extant species are considered.

In this paper, we present a statistical approach that resolves all of these issues. First, we use a broad phyletic sampling, ranging over 14 orders of magnitude in body size. Second, our statistical approach allows using only the number of extant species in a clade rather than phylogenetic or fossil information. Third, we derive the complete likelihood of the stochastic birth–death model, conditional on non-extinction of the clade and correcting for phylogenetic correlation, with allometric scaling of speciation and extinction rates, given data on extant diversity, clade age, and average body size of the species in the clade. The model is essentially the same as that of our predecessors [12,25–27] allowing easy comparison. In contrast to these studies, we find that the simplest macro-evolutionary explanation of the size-diversity pattern is a quarter power decrease of speciation and extinction rates with body size.

## 2. Methods

### 2.1. Data

We compiled data from numerous sources on the diversity, number of extinctions, age and mean body size of 198 Metazoan families in 61 orders, 11 classes and five phyla. The electronic supplementary material gives details on how we compiled these data, so here we only outline the key aspects. We included families (clades) that are considered to be monophyletic and for which the number of extant species per family (*N*_L_) is well known (based on biodiversity inventories). Where available, we also recorded the number of species that have become extinct per family (*N*_E_) based on fossil data (for extinctions that occurred millions of years ago), narrative reports in encyclopaedias (for extinctions that occurred in the last 2000 years) and the International Union for Conservation of Nature Red List of Threatened Species (for recent extinctions). These extinction data were not used for our estimation of speciation and extinction rates, but only to compare our predictions on numbers of extinction in order to assess the size bias of the fossil record. Because the fossil record is very incomplete for small-sized taxa, this led to a limited number of data points (see the electronic supplementary material). We recorded the evolutionary age (*T*) of each taxon (which we refer to as taxon age or clade age) from the fossil record and from published molecular phylogenies. When both were available, we used the larger value. This resulted in most taxon ages being based on the fossil record, and thus taxon age lies somewhere between stem age and crown age of the clade. Whether stem age or crown age is used is not crucial here, because we are not interested in absolute values of speciation and extinction rates, but only in their allometric scaling behaviour.

**Figure 1.**
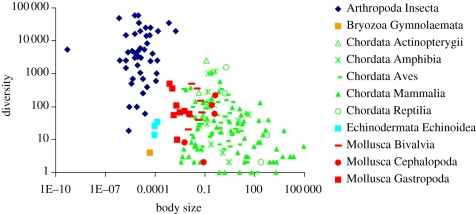
Diversity as a function of body size (in kg) in 198 families separated into 11 classes. The colour indicates the phylum the family belongs to.

**Figure 2.**
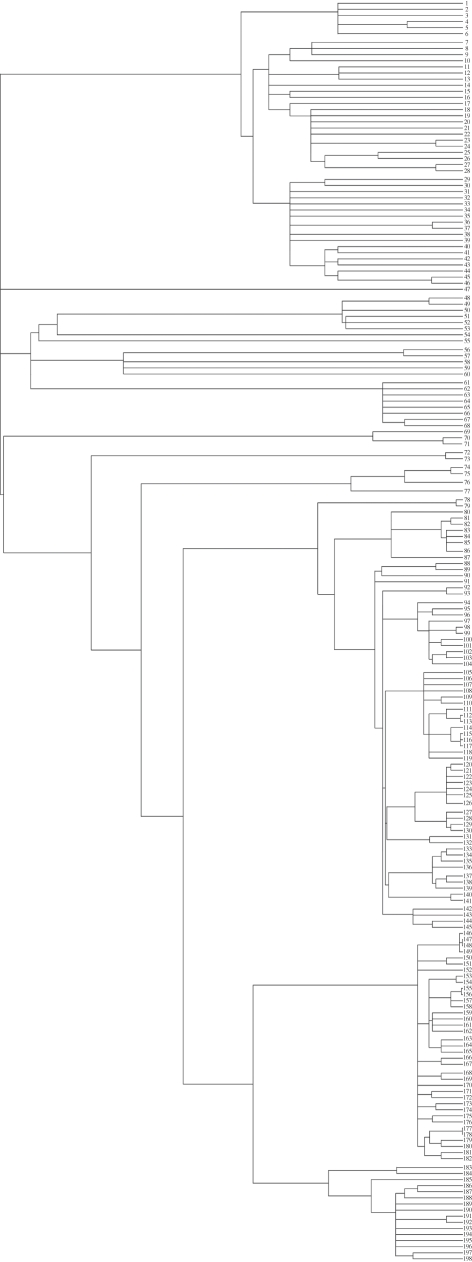
Supertree for the families used in this paper. The numbers correspond to the numbers in the data file in the electronic supplementary material.

The data are plotted by phylum in figure 1. We corrected for phylogenetic dependence (see §2.3) using a supertree that we built based on the fossil record [37]. Figure 2 shows the phylogenetic supertree. We refer to the electronic supplementary material for more details on compilation of the dataset and we provide a file containing the full dataset.

### 2.2. Model

#### 2.2.1. Macroevolutionary model

We used the stochastic birth–death model [38] for speciation (origination) events and extinction events which is the standard analytical model for studies of diversification [39]. It is described by a master equation for the probability ℙ[*N*_*L*_, *t*] of having *N*_*L*_ extant (living) species at time *t*, assuming a fixed speciation rate *S* and extinction rate *E* (we will return to this assumption in §4),2.1
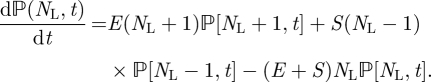
The initial condition is a single species at time *t* = 0,2.2
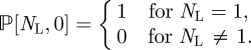
The solution, given by Kendall [38], is2.3
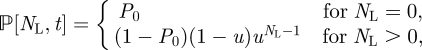
where2.4a
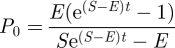
and2.4b
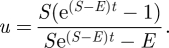


These parameters can also be written as2.5a
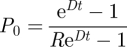
and2.5b
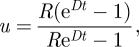
where2.6a
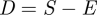
and2.6b
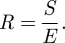
Here, *D* is the net diversification rate (resembling the net growth rate in population dynamics) and *R* is the diversification ratio (resembling the reproductive number in population dynamics).

In our meta-analysis, we (obviously) selected only those lineages that have at least one species that is currently still extant. This requires a correction for selection bias which can be done by conditioning on non-extinction of the clade [28,40–44]. So instead of (2.3), we use2.7
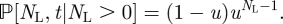


To compare our predictions with observations, it is useful to compute the expected number of extant species conditional on non-extinction. This is given by [44]2.8
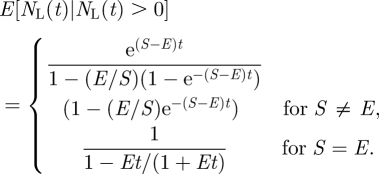


We also compared the prediction of the model for the expected number of extinctions *N*_*E*_ since the origination of the clade, conditional on non-extinction, with extinction data. The formula for the expected number of extinctions under the model is [44]


2.9
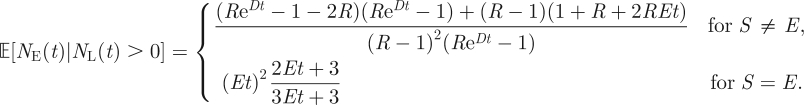


#### 2.2.2. Allometric scaling of speciation and extinction

The simplest non-trivial dependence of speciation and extinction rates that has a mechanistic basis in metabolism [36] is an allometric dependence (i.e. a power law),2.10a
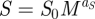
and2.10b
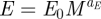
where *S*_0_, *E*_0_, *a*_*S*_ and *a*_*E*_ are parameters that will be estimated from data (see §2.3). This implies that the diversification rate2.11a
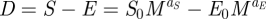
behaves allometrically only if *a*_*S*_ = *a*_*E*_. In contrast, the diversification ratio,2.11b
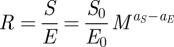
will always behave allometrically if *S* and *E* do, where the allometric exponent is the difference between the allometric exponents of *S* and *E*. Equations (2.10) assume a strict, deterministic, relationship between speciation/extinction rates and body size. We have looked at the consequences of relaxing this assumption by allowing noise in this relationship, but the results remain similar (see the electronic supplementary material and table 1).

**Table 1.** Maximum-likelihood estimates of the model parameters without and with (phylogenetically structured) noise in the allometric scaling relationships (2.10).

*S*_0_	*E*_0_	*a*_*S*_	*a*_*E*_
*without noise*			
2.3850 ± 0.2034	2.3834 ± 0.2037	− 0.24910 ± 0.0194	−0.24915 ± 0.0194
			
*with noise*			
2.4340	2.4340	−0.2218	−0.2217
2.1599	2.1607	−0.2169	−0.2169
2.1588	2.1578	−0.2169	−0.2169
2.1694	2.1677	−0.2171	−0.2171
2.1884	2.1869	−0.2071	−0.2071
2.2443	2.2420	−0.2327	−0.2327
2.2767	2.2757	−0.2291	−0.2290
2.2814	2.2794	−0.2193	−0.2193
1.9231	1.9208	−0.2275	−0.2277
2.3114	2.3088	−0.2336	−0.2337

Equations (2.5*b*), (2.7) and (2.10) form the full model, yielding the probability ℙ[*N*_L_, *t*|*N*_L_ > 0;*M*,*S*_0_, *E*_0_, *a*_*S*_, *a*_*E*_] of having *N*_L_ extant species of body size *M* at time *T*, conditional on non-extinction of the clade (*N*_L_(T) > 0) and assuming allometries for the speciation and extinction rates.

### 2.3. Statistical analysis

At first sight, assessing the allometry of diversification, speciation and extinction rates seems a straightforward two-step process. First, one estimates these rates for each family by maximum likelihood or method of moments, i.e. equating the expected number of extant (extinct) species with the observed number of extant (extinct) species [44–46]. Then, one regresses the logarithm of these rates against the logarithm of body size. However, as pointed out above, there are three problems with this approach. First, it involves two separate statistical methods (estimation of these rates on the one hand and regression on the other). Second, it cannot deal with *N*_L_ = 1, because the estimate of the net diversification rate then becomes 0, preventing logarithmic transformation. Solutions, such as leaving out the *N*_L_ = 1 data points or adding the arbitrary value of 1 to the rate before taking the logarithm, are *ad hoc* and, therefore, unsatisfactory (adding a different constant produces different parameter estimates). Third, the approach is unconditional, i.e., it is based on the unconditional expectation that follows from (2.3). The third problem can be remedied by taking conditional expectations [44], but the first two problems still remain. Therefore, we propose a different statistical approach based on likelihood maximization where the likelihood follows from the model described above.

#### 2.3.1. Maximum likelihood parameter estimation

The probability of each data point *i*, given the model parameters *Θ* = {*S*_0_, *E*_0_, *a*_*S*_, *a*_*E*_}, the clade age *T*_*i*_ and body size *M*_*i*_, and conditional on non-extinction of the clade is given by ℙ[*N*_L,*i*_(*T*_*i*_)|*N*_L,*i*_(*T*_*i*_) > 0;*M*_*i*_, *Θ*] according to (2.10). Therefore, the loglikelihood LL for this dataset is simply given by the sum over all *N* data points of the logarithm of these probabilities:2.12
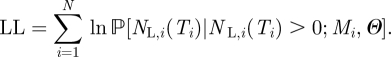


We performed likelihood maximization to find the parameters of this model, particularly the scaling parameters *a*_*S*_ and *a*_*E*_.

In deriving this loglikelihood, we have implicitly assumed that the allometries (2.10) are perfect. However, these relationships probably contain noise and this noise may be phylogenetically structured. This makes the problem much more complex. In the electronic supplementary material, we outline how we tackled this problem, but our results remain unaltered by adding this correction.

#### 2.3.2. Goodness of fit

We performed parametric bootstrap resampling to obtain goodness-of-fit measures (see [47]) and estimates for the errors in the parameters (1000 iterations). To compare the model just described with a model where there is no allometry in diversification (*a*_*S*_ = *a*_*E*_ = 0) we used the corrected Akaike Information Criterion, defined as2.13
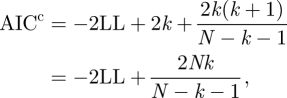
where *N* is the number of data points and *k* the number of parameters. One can then define model weights2.14
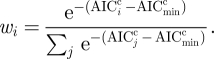


Because the model without allometric scaling of diversification (*a*_*S*_ = *a*_*E*_ = 0) is nested within the general model with allometric scaling, it is also possible to perform a likelihood ratio test. We report both the AIC^c^-values, the Akaike weights and the *p*-value of the likelihood ratio test.

#### 2.3.3. Allometric scaling of clade age

Although our method corrects for any dependence of clade age on body size by using the real clade ages, we were interested in the dependence of clade age *T* on body size. To assess this dependence, we assumed an allometric relationship2.15
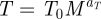
and estimated the parameter *a*_*T*_ by simple regression (after logtransformation of both clade age and body size).

## 3. Results

We concentrate on the results of the analysis without phylogenetically correlated noise in the allometric scaling relationships (2.10), because adding the noise did not change the results substantially. Our general conclusion is that both speciation and extinction rate decrease with body size with a quarter power-scaling exponent, and that this prediction is a surprisingly robust and good fit to the data.

**Figure 3.**
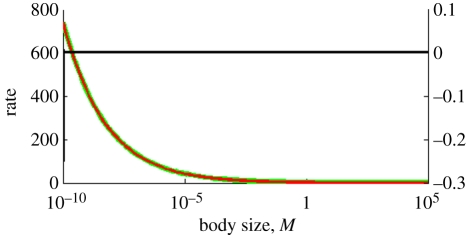
Predicted allometries for speciation rate (green, left axis), extinction rate (red, left axis) and diversification rate (black, right axis).

The allometric parameter estimates of best fit (table 1) indicate a significant body-size dependence for the speciation rate (*S*) and extinction rate (*E*) with a scaling exponent of around −0.25, but we find no such dependence for diversification (*D*) rate. Table 1 also shows the errors in the parameter estimates, obtained by parametric bootstrap. Because speciation decreases slightly more slowly with increasing body size than extinction does, the net diversification rate peaks around 10^−5^ kg (figure 3). However, this optimum is not noticeable in figure 3 and perhaps a spurious result because there are relatively few data points below it. Across the whole range of body sizes, the net diversification rate is very close to zero, thus for each body size speciation and extinction rates are remarkably similar (figure 3). This is in line with estimates based on well-studied taxa ([48], see discussion).

**Figure 4.**
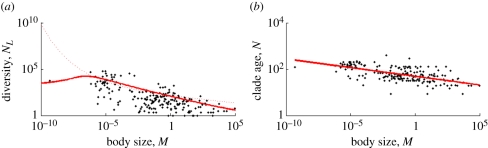
(*a*) The expected extant diversity as a function of body size (in kg) for two models (solid curve: fitted *a*_*S*_ and *a*_*E*_ and dotted curve: *a*_*S*_ = *a*_*E*_ = 0) and for the data of figure 1 (dots). (*b*) Data (dots) for Clade age (in million year) versus body size (in kg) and the fitted model (curve); the regression results were used to plot the curves in panel A, but this is only for presentation purposes; we used the actual clade ages for our statistical analysis.

Thus, the body-size dependence of speciation and extinction rates with an allometric exponent of −0.25 explains most of the observed relationship between diversity and body size (figure 4*a*); the remainder is explained by a small dependence of clade age (*T*) on body size (figure 4*b*) yielding an exponent of *a*_*T*_ = −0.08.

**Figure 5.**
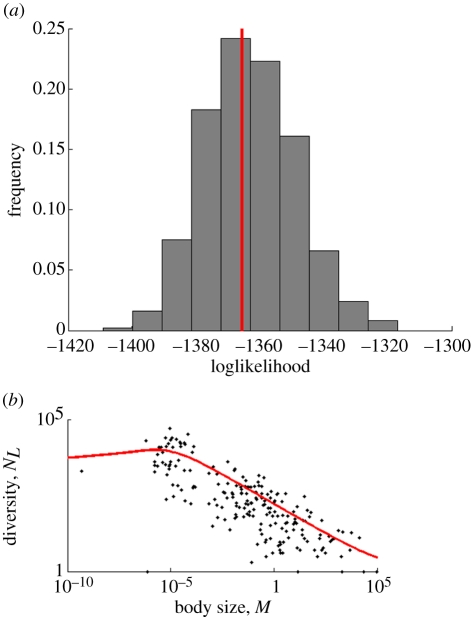
Analysis of model performance with simulated data. (*a*) Histogram of the probabilities of data simulated with the maximum likelihood parameter values. The line indicates the value for the real data. (*b*) A typical simulated dataset for extant diversity versus body size (dots) with the model prediction for this dataset (curve).

Even though the model is a simple model that, arguably, is not overly realistic (see below for further discussion), it fits the data surprisingly well. Figure 4*a* shows the extant diversity expected from the model with non-zero allometric scaling exponents (solid line) and for the alternative model with vanishing exponents, i.e. a model that assumes that the pattern is solely due to smaller taxa being of older evolutionary age (dotted line) as suggested by McPeek & Brown [27]. The model with allometries fits the data much better than the model without allometries (corrected AIC-values are 2739 versus 2823, giving rise to weights 1 and 0, respectively, and the likelihood ratio test gives *p* < 10^−16^). Not only does the model with allometries perform better than without allometries, it also gives a good fit in absolute terms, because the probability of the data is at the 46th percentile of the distribution of probabilities of data simulated (bootstrapped) with the ML parameter estimates (figure 5*a*), and the data are, therefore, consistent with the model (compare figure 5*b*, which shows a typical simulation dataset, with the real data of figure 4*a*).

To rule out the possibility that the similarity of the speciation and extinction parameters (i.e. resulting in *D* ≈ 0 across the whole range of body sizes) rests upon an artefact, we tested our predictions for robustness. We first determined whether there was a single point with a large leverage on the regression results (particularly the point with the lowest body size) by reanalysing the data after removing the data points one by one with replacement. This was not the case: the allometric exponents varied only between −0.26 and −0.24 with one exception of −0.21. We also reanalysed the data after removing data points one by one without replacement. When the order of removed data points was from small to high body size, we found hardly any effect until 27 per cent of the data had been removed, and when the order of removed data points was from large to small clade age, we found hardly any effect until 33 per cent of the data (clades older than 85 Ma) had been removed; removing all clade ages older than 60 Ma (thus avoiding the possibility that the major mass extinction event at the K–T boundary had any impact) yields quantitatively different parameters, i.e. a scaling exponent of −0.42, as can be expected because 45 per cent of the data points were removed this way, but it yields qualitatively still the same pattern: speciation and extinction both decrease with body size. As these percentages present a substantial reduction of the dataset, this suggests that our initial results are robust: clade age alone is an insufficient explanation for the data.

Our analysis with phylogenetically correlated noise in the allometric relationship (§2.2.2) yielded qualitatively and even quantitatively similar results with respect to the explanation of the body size–diversity pattern (table 1), i.e. that clade age alone cannot explain this pattern, and both speciation and extinction rates are predicted to decrease with body size with an allometric exponent of around −0.25. The optimization routine minimized the contribution of the noise term, suggesting that the speciation and extinction rates are well conserved and tightly linked to body size, or that the noise term takes a very different form than we assumed.

**Figure 6.**
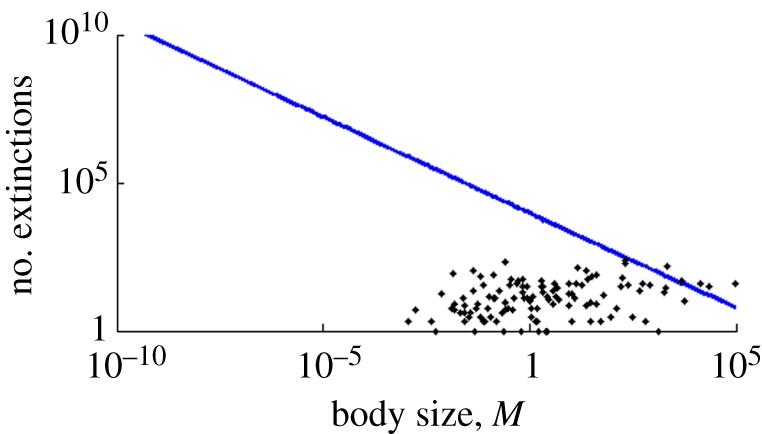
Expected (line) and observed (dots) number of extinctions as a function of body size (in kg).

The predictions for the expected number of extinctions are substantially higher than the number of extinction events inferred from the fossil record (figure 6), except for the largest body sizes. Even more interestingly, we predict that the expected number of extinctions (conditioned on survival of the clade) decreases with increasing body size. Because *S* ≈ *E*, the second expression of (13) applies, and we have 𝔼[*N*_E_(*T*)|*N*_L_(*T*) > 0] = (*ET*)^2^((2*ET* + 3)/(3*ET* + 3)) ≈ (*ET*)^2^ = *E*_0_*T*_0_*M*^2(*a*_*E*_^^+*a*_*T*_)^, so the expected number of extinctions is linear on a loglog plot, and has slope 2(*a*_*E*_ + *a*_*T*_) ≈ −0.66.

## 4. Discussion

The answer to the question ‘Can clade age alone explain the relationship between body size and diversity?’ is both no and yes. No, because we find that the size-diversity pattern is best explained by a significant decrease of speciation and extinction rates with increasing body size, even after accounting for the effect of clade age. Yes, because the estimated value of the allometric exponents of −0.25 suggests the following intriguing conclusion. Because generation time generally shows an allometry with exponent 0.25 [49–52], speciation and extinction rates expressed as events per generation (or millions of generations) scale as *M*^0^, i.e. they are independent of body size. This means that taxon age alone can indeed explain the size-diversity pattern, but only when expressed in generations. This suggests that speciation and extinction may indeed be life-history invariants [53].

Another interesting interpretation of our results is that both extinction rate and speciation rates decrease with decreasing population size (because larger organisms usually have smaller populations). This finding may have important consequences for models of community diversity, because the dependence of speciation on abundance can leave a clear signature on macro-ecological patterns [16,17,54,55].

In our analysis, we deliberately used the same standard constant-rate birth–death models as used in the aforementioned previous macro-evolutionary studies of the relationship between diversity and body size, because we can directly attribute the difference between our findings to our more coherent statistical approach and a broader range of body sizes in our data. However, we recognize that this null model of diversification [56] has some unrealistic features, the most prominent of which is the assumption of time-constant rates of speciation and extinction. This results in an exponential, unbounded, accumulation of species. In contrast, there is now considerable evidence for negative diversity dependence [57–61]. The proposed underlying mechanism is saturation of niches coupled with niche conservatism, or the cessation of niche construction (see [62] for the most mechanistic model to date). Also, it is known from the fossil record that diversity can remain constant for millions of years [63–65]. Notwithstanding this evidence, diversity-dependent diversification (or the ecological limits hypothesis) is not yet unanimously accepted [20,66,67]. Moreover, diversity increases with clade age in our dataset (see the electronic supplementary material), which is consistent with the constant-rate birth–death model [67].

Still, in future work it would be interesting to perform an analysis similar to that of this paper using the diversity-dependent model [61]. Incorporating such diversity-dependence is challenging and requires making further assumptions that may themselves be questionable. For instance, one would need to make assumptions on how the clade ‘carrying capacity’ depends on body size. Because this carrying capacity is most probably set by ecological factors, a relationship with body size (allometric or not) seems implausible. A better route for future research would be to use molecular phylogenies to estimate allometric scaling of speciation and extinction rates allowing the carrying capacity to be a free parameter (or perhaps to depend on range sizes or some other ecological variable). This will need to wait for the availability of phylogenies of many clades from a large range of body sizes.

There are other alternatives to time-constant speciation and extinction rates besides diversity-dependence. One could incorporate specific time-dependence speciation and extinction rates either directly or indirectly through time-dependence of body-size (e.g. [68]). This would require a mechanistic model for this time-dependence, or one could use a phenomenological description, based on observations, such as evidence for Cope's rule (the observation that the body sizes of the clade's species tend to increase over evolutionary time). Another alternative, which is one of our favourites, is the protracted speciation model, where speciation is allowed to be gradual rather than instantaneous ([69], see also [70]). In this model, speciation initiates at a constant rate and completes at a constant rate. One could study whether the rates of initiation and/or completion are dependent on body size. Protracted speciation is a viable alternative, because so far it is the only mechanistic model that explains the observed slowdown in lineage accumulation towards the present in a phylogeny of extant species [41,42] in contrast to the standard constant-rate birth–death model used in this paper or the diversity-dependent diversification model (see [61]) that typically lead to an upturn rather than a slowdown when extinction is non-zero (see [65,71] for counterexamples where such an upturn can be seen).

Given the simplicity of the model, its performance is surprisingly good. The fit to the data is very decent, both visually and in statistical comparison to the model without allometry of speciation and extinction. Our test with simulated data confirms this: simulations with the model yield *in silico* data that are similar to the real data, both visually (compare the real and simulated data points of figures 4*a* and 5*b*), and in a sound statistical comparison (the likelihood for the real data falls well within the distribution of the likelihoods of simulated datasets, figure 5*a*).

The parameter estimates are quantitatively consistent with independent estimates in the literature. The model, with the estimated parameter values, predicts that the speciation and extinction rates will be around 0.75 for a body size of 100 kg. Etienne & Apol [44], using counts of both extant and extinct species, reported rates for mammal clades that are similar to our predictions. Alroy [48], using fossil data, reported slightly lower values of 0.228 and 0.249 species per species per million year for the origination (speciation and immigration) and extinction rate, respectively, of North American mammals. However, because the absolute values of *S*_0_ and *E*_0_ are difficult to estimate, comparison of absolute values of speciation and extinction rates to literature values is not really informative. The ratio of *S*_0_ to *E*_0_ is more informative, and our finding of a close match between speciation and extinction rate agrees with Alroy's fossil estimates.

While this agreement with observations is encouraging, our prediction of the number of extinct species is higher than actually observed in the fossil record, particularly for small-bodied taxa (figure 6). But in fact, this prediction is in line with the general opinion [32] that more extinctions of small-bodied taxa have occurred than the fossil record tells us. This produces a bias towards larger taxa. Reasons for this are probably related to more complete preservation and easier discovery of larger-bodied organisms, and a research bias towards vertebrates.

There is one possible caveat. Although our test of robustness (re-analysis after removing part of the data) was passed successfully, the result that the net diversification rate is very close to zero across the entire body size range seems caused by the use of the constant-rate birth–death model combined with the strict allometries for speciation and extinction rate. If the net diversification rate were much greater than zero for some body size, the expected number of extant species would be very large. For example, for *S* = 0.5 and *E* = 0.4 and a clade age of 100 Ma, the expected number of species would be 10^5^ and the probability of substantially lower diversity values would be extremely low. The only way for the likelihood optimization routine to avoid such a scenario for all body sizes seems to choose the allometric relationships for speciation and extinction rates to be almost identical. This allows assigning appreciable probability to both low and high diversity values that we observe in the data. Figure 5*b* confirms this: simulations with the estimated parameters can produce both high and low diversities at the same body size value, e.g. *M* ≈ 10^−6^ kg.

While this caveat perhaps makes our results suspicious, the result *D* ≈ 0 may not be so unrealistic. An equilibrium approach to diversity seems natural [72], and in such an equilibrium, we have, by definition, *D* ≈ 0. Our results may be interpreted as an equilibrium being reached quickly after an initial radiation. Thus, the model with *D* ≈ 0 may perhaps be considered a proxy for diversity-dependent diversification. We can only verify this conjecture, once we have done an analysis with the diversity-dependent model.

While our analysis is by no means the conclusive explanation of the size-diversity pattern (if only because of the simplicity of the model), we do believe that we have cast considerable doubt on the argument that taxon age alone can explain this pattern. Instead, our results lend support to May's [11] conjecture that the evolutionary clock ticks faster for small organisms. This faster clock, combined with their older evolutionary age, can indeed explain their high diversity.
